# Non-Medicinal Treatment of Constipation

**Published:** 1903-11-21

**Authors:** 


### NON-MEDICINAL TREATMENT OF CONSTIPATION.

Although innumerable remedies have been suggested for the relief of chronic constipation, the successful treatment of it is still always a difficult problem. Dr. S. G. Gant[1] expresses the opinion that the failure to obtain good results is frequently due to the injudicious prescribing of purgatives, cathartics, and enemata when the costiveness is caused by fissure, polypi, hæmorrhoids, hypertrophy of the rectal valves, congenital malformations, benign or malignant stricture, or other disease of the rectum or colon which requires operation or local treatment. Again, in a very large percentage of cases the constipation is the result of improper diet, lack of tonicity of the bowel or insufficient exercise, and must be treated by correcting these conditions. It is in this class of cases, Dr. Gant remarks, that the physician is inclined to overdrug the patient and disregard entirely more rational methods which may be less satisfactory in the beginning, but which eventually effect a permanent cure. Dr. Gant has himself discarded for many years the use of drugs for the relief of constipation, and declares that he has treated by other methods several hundreds of patients suffering with this affection, in the majority of whom he has obtained a permanent cure, while the remainder have for the most part been to a greater or less extent relieved. The principal features of his non-medicinal treatment are the proper education of the patient, together with the administration of enemata when necessary, massage, the application of electricity, divulsion or division of the sphincter muscle, and division of the rectal valves—any one or all of these procedures being carried out as the case demands, the patient being warned that it may be several weeks before the desired results are obtained.

With regard to the proper education of the patient, Dr. Gant insists upon the necessity of temperance in manner of living, and regularity of habits. Meals should be taken at regular hours, and should be moderate in amount and eaten slowly. Fruits should be included in the dietary and should be eaten preferably in the morning or just before retiring. Water should be drunk freely throughout the day, and there are few more reliable laxatives than two or three glasses of cold or warm water taken in the morning on an empty stomach. Plenty of systematic exercise should be taken, in the open air if possible. A cold bath each morning is a useful adjunct. Dr. Gant points out that the administration of enemata daily to evacuate the bowel is a most pernicious practice and should be condemned. Early in the treatment of constipation enemata may be necessary, but their use should be discontinued as soon as other methods have become effective.

Massage is an essential feature of the non-medicinal treatment of chronic constipation. If possible a masseur should be employed as the patient cannot carry out the necessary manipulations himself without causing the abdominal muscles to contract. Massage should be commenced in the right iliac region, and should follow the course of the large intestine. This process should be gone through every other day at first, while later on twice a week will suffice. If the patient be unable to employ a masseur he may receive much benefit from daily rolling a cloth-covered metal or bowling ball over the course of the colon while lying in the recumbent position. Divulsion of the sphincter muscle is necessary when the muscle has become hypertrophied or irritable. Gradual divulsion may be accomplished without an anæsthetic by means of the passage of graduated bougies. Rapid divulsion is carried out with general anæsthesia, the muscle being stretched with the thumbs until its resistance has been overcome.

To perform the operation of valvotomy, Dr. Gant has devised a special clamp which can be fixed to the enlarged valve and left until it becomes separated by pressure necrosis. The clamp usually comes away in from four to six days. The advantages claimed for this method are its painlessness and freedom from hæmorrhage and other complications.

In concluding, Dr. Gant states that in his experience the results obtained from the non-medicinal treatment of chronic constipation are by far more satisfactory and permanent than those following the use of drugs.

[1] Medical Record, October 24.

